# Advances in Polymeric Colloids for Cancer Treatment

**DOI:** 10.3390/polym14245445

**Published:** 2022-12-13

**Authors:** Imran Ali, Sara H. Althakfi, Mohammad Suhail, Marcello Locatelli, Ming-Fa Hsieh, Mosa Alsehli, Ahmed M. Hameed

**Affiliations:** 1 Department of Chemistry, Jamia Millia Islamia (Central University), Jamia Nagar, New Delhi 110025, India; 2 Department of Chemistry, Faculty of Applied Sciences, Umm Al-Qura University, Makkah 21955, Saudi Arabia; 3 Department of Chemistry, Siddhartha Degree College, Aakhlaur Kheri (Saharanpur), Uttar Pradesh 251311, India; 4 Analytical and Bioanalytical Chemistry, Department of Pharmacy, University “G. d’Annunzio” of Chieti-Pescara, Build B, Level 2, Via dei Vestini, 31, 66100 Chieti, Italy; 5 Department of Biomedical Engineering, Chung Yuan Christian University, 200, Chung Pei Rd., Chung Li 320314, Taiwan; 6 Department of Chemistry, College of Science, Taibah University, Medina 46423, Saudi Arabia

**Keywords:** polymer colloids, colloidal polymeric nanomaterials, cancer diagnosis and treatment, future perspectives

## Abstract

Polymer colloids have remarkable features and are gaining importance in many areas of research including medicinal science. Presently, the innovation of cancer drugs is at the top in the world. Polymer colloids have been used as drug delivery and diagnosis agents in cancer treatment. The polymer colloids may be of different types such as micelles, liposomes, emulsions, cationic carriers, and hydrogels. The current article describes the state-of-the-art polymer colloids for the treatment of cancer. The contents of this article are about the role of polymeric nanomaterials with special emphasis on the different types of colloidal materials and their applications in targeted cancer therapy including cancer diagnoses. In addition, attempts are made to discuss future perspectives. This article will be useful for academics, researchers, and regulatory authorities.

## 1. Introduction

It cannot be denied that cancer has become a serious ailment from which millions of people have died globally. Worldwide, cancer has also been a cause of 9.6 million deaths and 18.1 million new cases in 2018 [1]. Consequently, we have to prepare ourselves more strongly to fight this lethal ailment. Among many approaches, chemotherapy is gaining importance as a way to treat different types of cancers [2,3,4,5,6] but it has been associated with several side effects. The most serious side effects are lymphedema, severe pain, fatigue, loss of appetite, swelling and drainage from the site of surgery, bleeding, infection, and organ dysfunction [7,8,9,10,11,12,13]. In addition, multidrug resistance (MDR) is another problem in chemotherapy. Some strategies have been developed and adopted by small-molecule inhibitors to control MDR. These strategies have been found to be of great importance in extending the effectiveness of chemotherapy and refining the clinical results of patients with cancers that are vulnerable to MDR development [14]. Even then, a hundred percent success could not be achieved in safe mode; therefore, there is a great need to develop safe and human-friendly chemotherapy. Nowadays, this disease has been included in the theranostic administration of numerous ailments. To the best of our knowledge, J. Funkhouser was the first to name the term theranostic in a press release. This term was used for the description of investigation and treatment methods in a sole part. Theranostics means treatment using imaging, diagnostic, and treatment of cancer. It helps too much to control cancer treatment. It also helps in drug selection, drug dose, and delivery to the exact position (target) in the human body [15]. In addition, the contribution of such type of management to the economy of a country cannot be ignored because it reduces the cost with precise and effective drug processes. Since this treatment protocol is based on a target and, consequently, the least side effects are observed. This approach is represented in this article using polymeric colloids.

As stated above, there are severe side effects of chemotherapy, which compel scientists to explore new approaches [16]. Thus, a theranostic approach could be very helpful for not only cancer treatment but also for gaining efficiency of persistence. The treatment of cancer needs more advanced techniques to analyze cancers; however, nanotechnology showed good results in the treatment of numerous cancer [17]. Nanotechnology enabled new methods of imaging and therapy which are successfully being used in everyday clinical practice. Inspection of nanoparticles-centered imaging and therapy is covering a large area day by day. Nanotechnology has become an advanced tool in not only the identification but also in the delivery of drugs. Probably, nanotechnology will participate significantly to achieve the required task in modified drugs and management. Cancer theranostics involves metallic nanoparticles, polymeric nanoparticles, liposomes, carbon nanotubes quantum dots, dendrimers, etc. in nano-formulations. Among many systems, polymer colloids are attracting scientists because of their many useful properties [18]. Basically, a polymer colloid is a dispersal of submicron particles of the polymer in a liquid (generally aqueous) medium. Among many methods, emulsion polymerization is the most widely used. The remarkable features of polymer colloids in drug development are high capacity in terms of drug loading, biodegradability and biocompatibility, and ease of preparation [19]. If the concentrations of micelles decrease, the micelles in the colloidal solution decompose. It is because the formation of micelle and colloidal solution occurs at only high concentrations. This thing can also be considered as a limitation of the micelle-forming copolymer if they are used as a drug in cancer therapy. Some reviews are available on this subject using classical polymers [20,21] but polymer colloids-based review for cancer diagnosis and treatment is not found. Therefore, efforts are made to review the advances in polymer colloids in cancer treatment. The current article describes the state-of-the-art of polymer colloids in the theranostic management of cancer, as well as highlights the future challenges and perspectives regarding such a type of research area.

## 2. Polymer Colloids

Polymer colloids are liquid-phase dispersions of polymer particles; often water. The diameter of a particle can range from 10 to 1000 nm, and each particle typically (but not always) comprises a large number of distinct polymer molecules. Some colloidal polymers are easily generated from naturally occurring components or occur naturally. Typically, emulsion polymerization is used to create functionalized polymer colloids. Monodisperse polymer colloids with acetal, aldehyde, chloromethyl and amino functionalities are prepared using a multi-step emulsion polymerization technique. The lipophilic drugs have solubility in the oily core or polymer matrix of polymers are more readily combined than hydrophilic molecules. The polymer colloids may be of different types such as micelles, liposomes, emulsions, cationic carriers, and hydrogels [19]. Therefore, the uses of these polymer colloids in cancer treatment, diagnosis, and management are discussed in the following sub-sections.

### 2.1. Micelles in Cancer Treatment

At low concentrations of the ingredients, micelles are not formed; however, they are formed if the concentration exceeds a certain threshold value. The concentration at which the first micelle appears is known as critical micellization concentration (CMC). In such a type of solution, micelles behave as colloidal particles because of gaining colloidal range. The solution is termed a colloidal solution. The ingredients present are called associated/colloidal ingredients. A polymer can also be considered a colloidal polymer if it forms a colloidal solution by attaining a colloidal range/size at CMC. The micelle gives rise to heterogeneity in the solution, and polymeric colloids represent a heterogeneous system. 

The use of the theranostic approach in diagnosis, drug delivery, and cancer therapy has recently been a subject of great interest [22]. The size of the colloidal polymers is in the range of 10–1000 nm and they cannot enter the human cell normally. This involves many cellular interactions including antibodies, receptors, and enzymes [23,24]. Nanotechnology can be used to make them ideal candidates for cancer treatment [25,26]. The literature indicates the formulations of several nanoparticle theranostic agents. Different materials such as Au, Si, C, etc. [27,28], have been used for many nanoparticle theranostic agents. These materials gave good results when tested for early cancer detection but immunogenicity, toxicity, and low clearance value from the body were the main disadvantages linked with these materials [29,30]. Some of the macromolecules used for preparing NPs include polylactic acid (PLA), poly(β-caprolactone), poly(lactide-co-glycolide) (PLGA), poly(alkylcyanoacrylate), and polyglycolic acid [31]. When the concentration of these NPs is increased, they combine to make polymers and after that, they attain a colloidal range. Moreover, synthetic polymeric biodegradable nanoparticles were also explored with the help of computational evaluation [32,33]. Some of the biodegradable colloidal polymers include poly(L-aspartate), poly(2-hydroxyethyl-L-aspartamide), poly(D,L-lactic acid-co-glycolic acid), poly(ethylene glycol) (PEG), poly(ε-caprolactone), poly(*N*-vinyl pyrrolidone) (PVP), poly(hydroxypropyl methacrylamide) (PHPMA), poly(*N*-isopropyl acrylamide) (PNIPAM), poly(ethylene glycol), poly(methyl methacrylate), poly-(chloromethyl-styrene) (PCMS) etc. 

**Figure 1:**
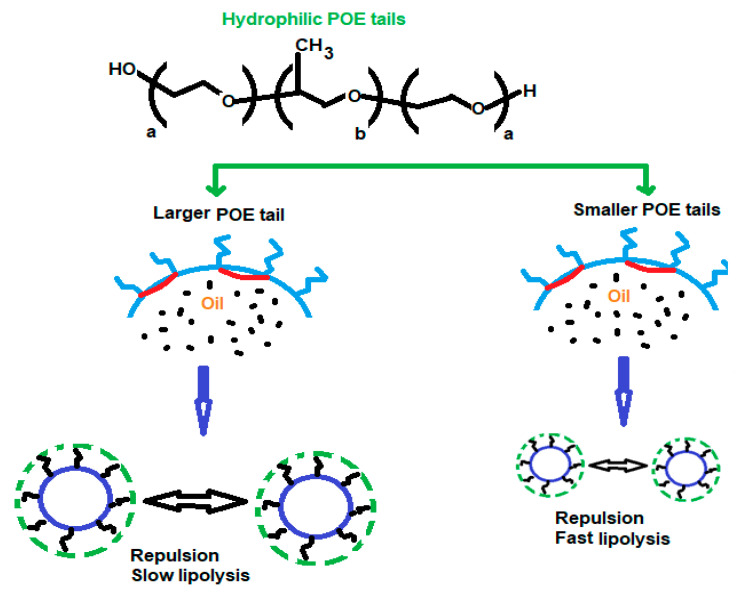
The influence of the poloxamer structure on the interfacial and macroscopic properties of nano-emulsions [35].

**Figure 2:**
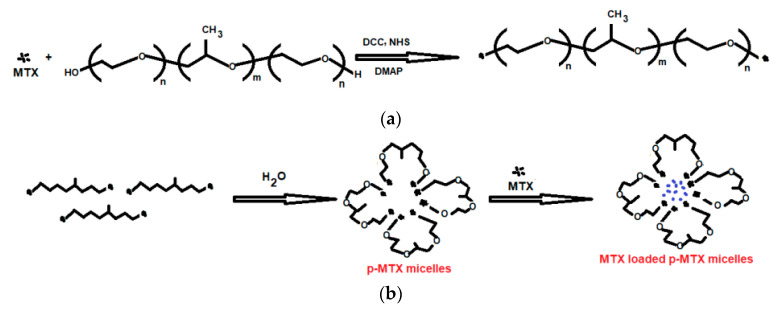
Synthetic Route of (**a**): p-MTX conjugates and (**b**): the formation of p-MTX micelles and MTX-loaded p–MTX micelles [40].

**Figure 3:**
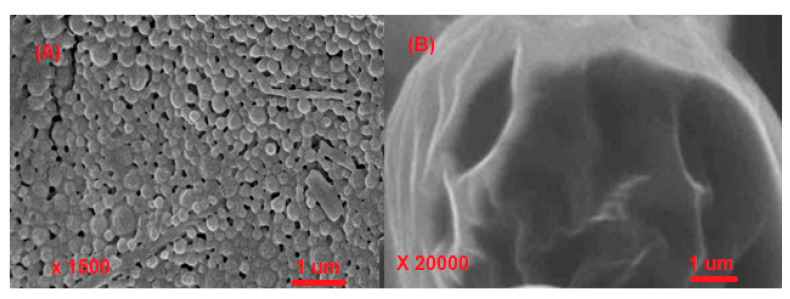
FESEM images of docetaxel-loaded PLGA–TPGS/Poloxamer 235 nanoparticles and microparticles. (**A**) nanoparticles; (**B**) microparticles [41].

Pluronics consist of propylene oxide fragments and ethylene oxide which are hydrophobic and hydrophilic, respectively. A structure of (PEO)a-(PPO)b-(PEO)a type [34] was obtained using the poly(ethylene oxide), PEO, and poly(propylene oxide), PPO. The applicability of the synthesized structure was just because of their self-gathering for the formation of micelles. In addition, not only emulsification but also protection of nanocarriers is also done. In emulsification, the nature of emulsion is very important in pharmaceutical sciences. If the water-in-oil emulsion is to be prepared, a poloxamer with short hydrophilic tails and a long hydrophobic block (with low HLB) are taken. It stabilizes water-in-oil emulsions according to the Bancroft rule. On the other hand, if the oil-in-water emulsion is to be prepared, a poloxamer with longer hydrophilic tails and a shorter hydrophobic block are taken. Hence, the POE/POP ratio determines the nature of the synthesized emulsion as shown in (Figure 1) [35]. A polymer of lactic acid (PLA) has been approved by FDA. This type of polymer has attracted many researchers. In paclitaxel, methoxy poly(ethylene glycol)-poly(lactide) copolymer acted as a synthesizer of micelle-based polymer. Nowadays, a formulation of vitamin E-TPGS and mPEG-PLA based on micelle is being used for paclitaxel delivery for an increment in Genexol-PM effectiveness against MDR cancer [36]. Another anti-cancer drug that has taken an important place in anti-cancer drug development is PEGylated polymer of caprolactone (PEG-PCL). Nanoparticles based on PEG-PCL micelle target cancerous sites. A star-shaped folate-PEG-PCL copolymer was prepared by Cuong et al. [37] for the encapsulation of doxorubicin for the directed distribution in breast cancer. For this purpose, folic acid was used for the modification of the PEG series. Overexpression of the receptor of folate was also observed in both breast and ovarian cancers. Therefore, folic acid conjugation in the carrier of the micelle (PEG-PLA) [37] played an important role in the transport of the medicine at an exact site. A polymer of lactic-co-glycolic acid (PLGA) has also taken a place among the polymeric nanocarriers just because of its (i) biocompatibility, (ii) biodegradability, and (iii) poor water solubility. These properties made this polymer a bioavailable polymer. The use of PLGA in the anti-cancer formulation has been approved by the US FDA. The toxicity level was found minimum in the drug delivery system when PLGA was used. Commercial PLGA polymers are available in diverse molecular masses and alignments that affect their biodegradation, which involves too much time variation [38]. The formation of PEG-poly(amino acids) co-polymeric-based micelles involves the polymerization of amino acids for the formation of a hydrophobic core. ADR-conjugated PEG-P(Asp) molded micelle-based construction in the nano-size range was confirmed in vitro anti-tumor action. In another study, the same group studied the influence of a PEG block on the conformation of a poly(β-benzyl-L-aspartate) segment in organic solvent [39]. In addition, other polymeric materials such as MTX-loaded p–MTX micelles [40] (Figure 2), and docetaxel-loaded PLGA–TPGS/Poloxamer 235 nanoparticles and microparticles were also synthesized to use in cancer therapy [41] (Figure 3). Daraba et al. [42] synthesized hydrophobic poly(ε-caprolactone) (PCL) and hydrophilic cross-linked poly(vinylpyrrolidone) (PNVP) polymeric nanoparticles. The authors used these carriers to anchor cisplatin via emulsion polymerization. Furthermore, the authors studied the drug release from these two nano-polymeric carriers and observed PNVP as a high release of the drug than PCL carrier. Furthermore, these two studied carriers were found to show good compatibility with the blood during hemolysis. The authors used MCF-7 and A-375 cancer cell lines to test the anti-cancer activities of loaded drugs and no toxicity was observed with the two studied carriers.

The micelle-based materials acting as nanocarriers are summarized in Table 1.

### 2.2. Liposomes in Cancer Treatment

The importance of liposomes cannot be ignored in cancer treatment. Enzyme-responsive liposomes have played an important role in the delivery of anti-cancer drugs [50]. First of all, liposomes were reported as drug carriers in 1971 by Gregoriadis et al. [51]. Since then many papers have been published in this area. Spherical vesicles-type liposomes have both hydrophilic and hydrophobic cores [52]. This property helps the liposome in the capsulation of hydrophilic and hydrophobic drugs [53]. In addition, other properties such as biocompatibility, biodegradability, flexibilty, and reduced side effects have also made these liposomes wonderful drug deliverers [53]. A PEGylated liposomal formulation (Doxil) was used by Johnson & Johnson for the encapsulation of an anti-cancer drug [54]. In 2011, a study regarding the imbalance between the demand and supply of Doxil was performed [55]. It was observed that the manufacturing unit was temporarily shut down due to some quality control issues [56]. A list of liposomal-based anti-cancer drugs is summarized in below Table 2.

### 2.3. Emulsions in Cancer Treatment

The emulsion can also be applied for cancer treatment. Emulsion-based formulations of drugs are mostly applicable for topical or transdermal uses [58]. The emulsion is a type of colloidal solution that has an oil and aqueous phase [59]. The drug exists in the solubilized form because it solubilizes both types of drug, i.e., lipophilic and hydrophilic [60]. Nowadays, nano-emulsion and microemulsions are being used in drug delivery systems. Microemulsions loaded with gemcitabine [61] have been used against many solid tumors and are also used in colorectal cancer treatment [62]. The most attractive point regarding drug-loaded microemulsions is their high hemolysis activity (17–21%). The authors studied %hemolysis of micro-emulsion solutions 10 μL of 1 mg/mL of GEM loaded in micro-emulsions and 10 μL of 1 mg/mL of GEM dissolved in water. These results indicate variable %hemolysis. Fofaria et al. [63] described nano-emulsion formulations of piplartine anti-cancer agents. The authors reported a 1.5-fold increase in bioavailability of the piplartine after encapsulation. Furthermore, the authors claimed this nano-emulsion as a delivery method for oral intake of piplartine, which improved the oral bioavailability, solubility, and anti-tumor efficiency.

### 2.4. Cationic Carriers in Cancer Treatment

Anti-cancer drugs can also be delivered through ionic polymers. The ionic forms of anti-cancer drugs are loaded on to cationic polymers. The developed formulations have positive surface charges with robust cellular interaction features and good cellular acceptance. Chitosan has been used as a carrier in cancer treatment due to its anti-cancer effects [64]. Chitosan has been shown to cause apoptosis and bladder tumor cell death via caspase-3 activation [64]. Polymethacrylates have vinyl-base (cationic polymers) showing the capability to form polyplexes on the polynucleotide condensation. These molecules have a wide range of chemical structures with varied molecular weights [65]. The gene delivery efficacy has been increased by balancing between lysine moieties and free amino groups. This resulted in DNA complex formation with imidazole heterocycles; responsible for endosomal escape [66]. A commercially obtainable cationic polyamine of polyethylene imine is one of the most effective and extensively studied cationic polymers [65]. PEI was shown to interact with serum proteins of negatively charged erythrocytes. This precipitated in vast bunches and stick to the cell surface [67].

### 2.5. Hydrogels in Cancer Treatment

The hydrogels have attained a special position in anti-cancer drugs deliverable molecules because of their low toxicities [68]. The numerous chemotherapeutic drugs have been anchored in hydrogels and rooted near the tumors to obtain high drug amounts in tumor tissues for an extended period. In addition, self-healing hydrogels have been extensively utilized for tissue regeneration and engineered owing to their outstanding biocompatibility and connections to nearby tissues. The importance and demand of self-healing hydrogels for in situ transport of drugs are rising [69,70,71]. The hydrogels are good carriers for drug co-loading due to their protective properties and suitable injections. Kim et al. [72] reported the loading of doxorubicin and 5-fluorouracil on a hydrogel as a carrier. The authors reported sustainable drug delivery until 18 days later. The 5-fluorouracil anchored on pluronic hydrogel or 5-fluorouracil-loaded diblock copolymer hydrogel was mixed with DOX-loaded microcapsules to prepare two drug formulations. Both the formulations showed enough variability in vitro for injection easily into tumor and gel in situ at body temperature. Lerouge et al. [73] described hydrogel and used it for the encapsulation and propagation of T lymphocytes. This led to released interferon-γ (IFN-γ), cytotoxic biomarkers, and induced annexin V appearance through incubation with tumor cells.

Some polymer colloids-based anti-cancer drugs under clinical trials are summarized in below Table 3.

## 3. Targets in Cancer Therapy, Drug Delivery, and Controlled Release

The identification of the target is the most important step to understanding the mechanism of action. A proper selection of ligands depends upon the site affinity, which is supposed to hit. Such receptors have been termed the targets of the drugs/ligands. In cancer therapy, folate, glycoprotein, transferrin, and epidermal growth factor receptors (EGFRs) are the most widely considered receptors [82]. Transferrin transfers iron into proliferating cells through the blood by attaching to its receptor. Once the transferrin is adopted, the release of iron occurs to cause endocytosis in the cellular acidic medium. The receptor of transferrin is accountable for the homeostasis of iron and cell growth regulation [83]. Thus, the over-expression of such types of proteins in metastatic and the resistance were shown by the cancerous cells against the drugs, as compared to the standard cells. It is due to an increased concentration of iron; forming this receptor as a relevant receptor in cancer treatment [40,42,54]. On the other hand, the receptor of folate is a 38 kDa glycosyl-phosphatidylinositol conjugated glycoprotein. It is considered a tumor marker investigated by many scientists. Such a type of receptor fixes not only folic acid but also the conjugates of their drugs. Moreover, the fixation of nanocarriers of folate-anchored with a high affinity was also noted [47]. This type of receptor causes a fixation by internalizing into the cells via endocytosis [47]. In addition, folic acid is essential for nucleotide-based synthesis, *viz,* adenine, guanine, cytosine, and uracil. Moreover, normal cells do not allow the passage of folate-conjugate, but only a reduced form of folic acid is transported, i.e., 5-methyl-tetrahydrofolate [84]. The entry of the folate conjugate into the cancer cells happens mainly via the folate receptor due to the upregulation of these receptors on cancerous cells as compared to normal cells [85]. A wide range of tumor over-expression of receptors of folate includes different kinds of cancers. The additional point in the property of folate ligands is their inexpensiveness, nontoxicity, and non-immunogenicity. Moreover, they showed a high-binding affinity, constancy in storage and movement, and are bondable with receptors of nanocarriers epidermal growth factor [86]. The EGFRs, family members of receptors of tyrosine kinase, are extremely upregulated on the surface of cancerous cells. The EGFR binds to six known endogenous ligands: EGF, transforming growth factor-α, amphiregulin, betacellulin, heparin-binding EGF, and epiregulin [87]. EGFR activation has occurred through one of these ligands. This activation excited signaling processes within the cell, which were involved mostly in cancerous evolution and progression [36,44]. The over-expression of EGFR occurred mainly in breast, lung, colorectal, and brain tumors [88,89,90,91]. Glycoproteins lectins are the types of proteins that can recognize and assign precisely to the glycoproteins; expressed on the surface of cancerous cells. The expression of glycoproteins on cancerous cells is found different from that of ordinary cells. In addition, lectin-based targeting has also been useful in targeting colon tumors. The cancerous growth can be prohibited by checking the construction of new vessels of blood in the cancerous core. This results in a low or no blood supply, hindering the delivery of oxygen and other nutrients. Thus, nanocarriers designed to attack angiogenesis can prove to be very beneficial for controlling cancerous progress and related metastatic potential [59]. The tumor targeting has the succeeding qualities: (i) no need for the nanocarriers to reach their target site; (ii) the comfort of convenience to fix to endothelial receptors post-intravenous injection; (iii) endothelial cells are less prone to the risk of evolving conflict to treatment than cancerous cells; and (iv) this tactic applies to all types of cancer because of the expression of the markers on VEGF receptor of endothelial cells. The vascular endothelial growth factors (VEGFs) encourage cancerous angiogenesis and neovascularization by their capabilities to fix and stimulate the VEGF receptor (VEGFR) signaling cascade [9]. The most broadly explored receptor is VEGFR-2 among the VEGF receptors. The inhibition of angiogenesis can be conducted by directing VEGF to stop ligand binding to VEGFR-2. Another method to conduct the same direction is the attack on VEGFR-2 to decrease VEGF binding and trigger an endocytic pathway [92].

Micelles have abilities to exhibit sustained release, but these require specific properties. For example, micelles must not lose stability on dilution and low chain mobility core properties [93]; however, physically entrapped drugs in micelles have low diffusion coefficients to succeed in a continued discharge profile [10]. Pluronic-PAA (poly(acrylic acid)) micellar formulations have also been used in many oral drug delivery systems because the specificity of polymeric configuration meets all the requirements for actual oral drug delivery [10]. The latter is projected to improve the gathering of nanocarriers in the targeted tissues. It instantaneously increases the selective uptake via endocytosis mediated with the receptor. An exact target selection plays an important role in cancer diagnoses. Targeting may be based on carbohydrate moieties, monoclonal antibodies (mAbs), peptides, and aptamer [10]. In addition, the destabilization of the micelle mixed with PLLA/PEG was found to be pH-dependent. In conjugation with folic acid, the mixed micelles were found to be more effective in cancer destruction because of quicker drug release and tumor uptake mediated with the receptor of folate [94].

In biomedical engineering, protein and other materials (based on peptides) have gained the attention of many scientists just because of their applications in drug delivery systems. Their biophysical and biochemical properties have made protein-based material a better drug releaser [95]. Keeping many properties into consideration, many synthetic drug delivery-based materials are being synthesized. These are synthesized by considering the biocompatibility, purification, scalability, tuneability, and less toxicity features [96]. On the other hand, many natural biopolymers are under clinical trial and being studied continuously; for example, keratin, silk, collagen, albumin, elastin, gelatin, and resilin [97].

Polymeric materials have many types; some of them are pH dependent, i.e., their function show variations in the mechanism involving the attachment with the target; if pH of the medium is changed. These polymers are termed pH-responsive polymers (PRPs). The activities of the surface, chain conformation, and solubility change may be tuned by changing pH of the solution. PRPs are of two types: (i) natural PRPs such as alginic acid, hyaluronic acid, chitosan, heparin, and cellulose derivatives, and (ii) synthetic PRPs such as poly(histidine) (PHIS), poly(L-glutamic acid) (PGA), and poly(aspartic acid) (PASA). These are biocompatible, degradable, and pH-sensitive polymers [98]. We could not find any paper on polymer colloids as drug carriers; describing the effect of pH and temperature on their stability; however, it is important to mention the effects of pH and temperature on the stabilities of polymer colloids. Consequently, we selected some papers, which are discussed in this paragraph. Ishikawa et al. [99] studied the effect of pH on the stability of aqueous polymeric dispersions. The zeta potential was slightly influenced by altering the pH values. The polymers were stable in a wide range of pH, however, the zeta potential was altered with changing pH. In addtion, Jaquet et al. [100] studied the effect of pH on the stability of poly-acrylic acid brushes on polymer colloids. The authors reported that poly-acrylic acid chains were pH-sensitive with alterations in hydrophobicity, charging, conformation change, and polarity. Wojciechowski et al. [101] studied the colloidal stability of styrene and acrylic copolymers in the presence of TiO_2_ and CaCO_3_. The authors studied the stability at pH 8.2 for CaCO_3_ and 7.5 for TiO_2_. The colloids were found to be stable. Aseyev et al. [102] reviewed the temperature depending on the stabilities of various polymer colloids in water. The reviewed polymer colloids were poly(vinyl methyl ether), poly(*N*-vinylcaprolactam), and poly(*N*-isopropylacrylamide). As per the authors, the temperature stabilities varied from one polymer to another. Later on, Korshak and Vinogradova [103] reviewed the effect of temperature on the stabilities and chemical structure changes of the polymers. The authors reported changes in the stabilities and slight structural alterations at different temperatures. Recently, Hu [104] also reviewed the effect of temperature on the stabilities of the polymers. The author emphasized the advances in polymeric phase alteration. Some changes were observed in the polymeric phase alterations. In this way, it is clear that polymeric colloids are susceptible to pH and temperature. Therefore, the effects of pH and temperature on the drug loading of the polymeric colloids should be studied for publications, patents, or clinical trials.

**Figure 4:**
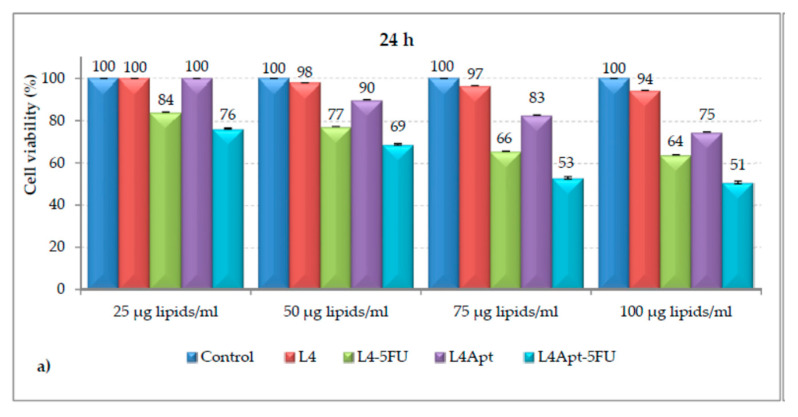

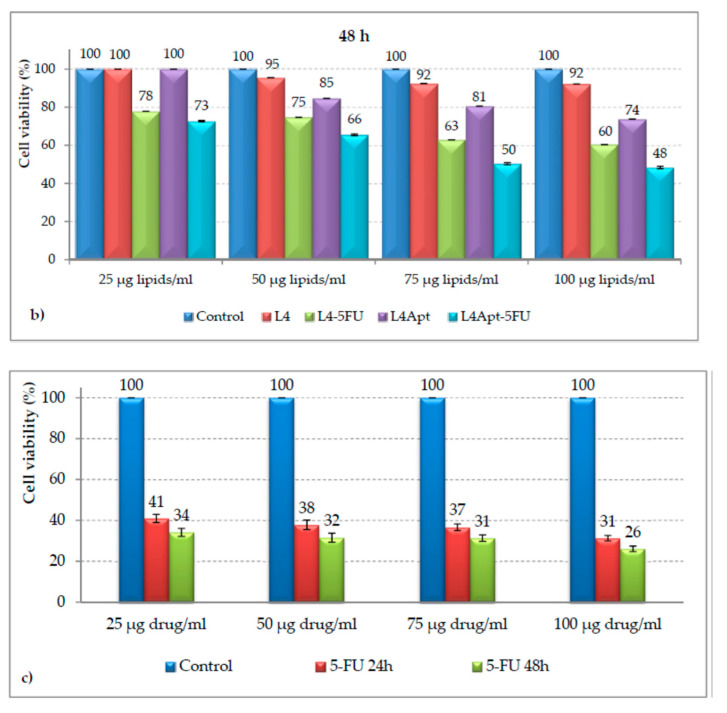
The cell viabilities of TE 354.T with L4, L4-5FU-15, L4Apt, L4Apt-5FU-15 with 25, 50, 75, and 100 µg lipids/mL doses for (**a**): 24 h and (**b**): 48 h and (**c**): 5-FU with 25, 50, 75, and 100 µg drug/mL doses for 24 and 48 h [106].

**Figure 5:**
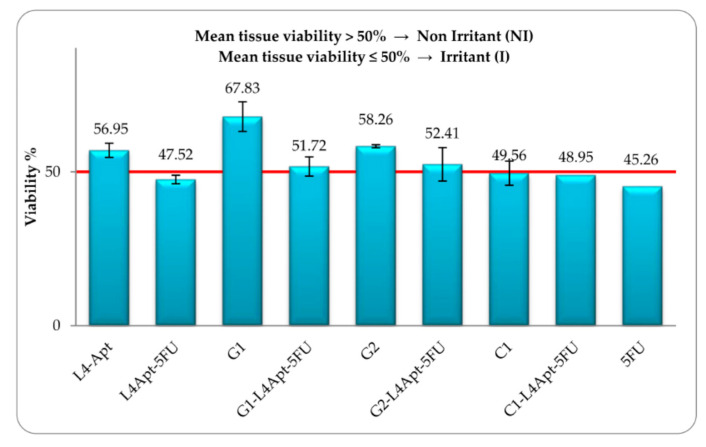
The in vitro cell viabilities of the different formulations in SkinEthic™ RHE tissues [107].

Targeted chemotherapy is gaining importance due to its less or no side effect. Many papers have been published on this issue using various nanoparticles as carriers, but we are relating them to polymer colloids. Rață et al. [105] prepared carboxymethyl chitosan nanoparticles which were functionalized with poly(*N*-vinylpyrrolidone-alt-itaconic anhydride) anchored with 5-Fluorouracil and AS1411 aptamer. The authors studied the release of 5-fluorouracil and observed its diffusion via the polymeric membrane. Furthermore, the authors reported outstanding hemocompatibility with no toxicity on the MCF-7 cell line. Cadinoiu et al. [106] used functionalized AS1411 aptamer liposomes for targeted therapy. The authors studied in vitro release of 5-fluorouracil and reported a low growing quantity of the released. The basal cell carcinoma TE 354.T cell lines were used for in vitro cell viability, apoptotic effects, and targeting ability. The cell viability of TE 354.T cells incubated with L4, L4-5FU-15, L4Apt, L4Apt-5FU-15, and 5-FU are shown in Figure 4. As per the authors, the functionalized liposomes were more effective than non-functionalized liposomes. Furthermore, the same group [107] reported a sustainable release of 5-fluorouracil to the tumor site by using liposomal drug formulations with AS1411-aptamer. The in vitro study was carried out to evaluate the penetration efficiency of 5-fluorouracil via the strat-M membrane, and the efficiency of cytostatic activity. The optimal liposomal formulation was found to be a crosslinked gel of sodium alginate and hyaluronic acid with AS1411-aptamer conjugated liposomes anchored with 5-fluorouracil. This was found to have biosafety effects, which can be utilized as a new approach of therapeutic for basal cell carcinoma. The in vitro cell viabilities of the different formulations in SkinEthic™ RHE tissues are shown in Figure 5. In another study, the same group [108] developed topical gel formulations using hyaluronic acid and sodium alginate having AS1411 aptamer; functionalized polymeric nanocapsules anchored with 5-fluorouracil for treating skin cancer. This formulation was found to have good permeability of 5-fluorouracil and be non-irritating to the skin. The cytotoxic study on TE 354.T cell lines indicated good cytotoxicity. As per the authors, the developed formulation was found to have good biosafety and anti-tumor properties; an attractive skin cancer treatment approach.

The stimuli-responsive drug delivery is gaining importance and polymeric nanocarriers are the most promising regimens for malicious cancers. This approach includes several advantages such as improved therapeutic involvement and reduced toxicity. Zhang et al. [109] described the use of polypeptide poly(l-histidine) as an appropriate polymer for the drugs delivery. The authors described the state-of-the-art design and creation of pH-sensitive nanocarriers based on this polymer. In addition, the authors highlighted the future challenges and perspectives of this material. The same group [110] also presented a review article on porous organic polymers highlighting the importance of these polymers due to their flexible morphologies, ordered pore arrangements, and tunable biological features.

Some FDA-approved nanomedicines for the treatment of cancer are summarized in Table 4.

## 4. Cancer Diagnosis

The detection of cancer at an early stage is very essential to treat this ailment appropriately. Hence, one of the most important approaches to managing cancers is the early-stage diagnosis. As per Barash et al. [112], only 16% of lung cancer is diagnosed in the localized phase; a curable stage. Not only the type of cancer but also its position as well as its size determine the cancer diagnosis. The commonly used ways for cancer diagnoses include positron emission tomography scan, biopsy, computed tomography, biosensors, magnetic resonance imaging, X-ray, fluorescent imaging, radionuclide, etc. The above modalities have also been coupled with nanotechnology for an early-stage detection of cancers. This part deals with a polymeric colloidal material-based cancer diagnosis, which attains a colloidal range in the solution. Numerous nanoparticles have been employed for cancer detection but the most widely used are polymeric colloidal materials just because of their biodegradability and ability to carry several molecules that detect lesions. In addition, polymeric colloidal materials with targeting moieties display a beneficial medication with an imaging mediator to be predictable to take an outstanding theranostic stand. Moreover, polymeric colloidal materials may openly convey the imaging mediator to the lesion and can be controlled in many ways such as oral, inhalation, or intravenous. For the diagnosis of cancer, many identities are used, i.e., tumor necrosis factor-alpha (TNF-α), α-fetoprotein (AFP), antigen 125 (CA125), cancer antigen 153 (CA15-3), cancer antigen 19-9 (CA19-9), epidermal growth factor receptor (EGFR), carcinoembryonic antigen (CEA), breast cancer (BRCA), human epidermal growth factor receptor 2 (HER2), interleukins (ILs), prostate-specific antigen (PSA), mucin 1 (MUC 1), vascular endothelial growth factor (VEGF) and squamous cell carcinoma antigen (SCC-Ag) [113,114]. Usually these have played an important role in the transport of imaging and molecules of therapy into the tumor site.

For the diagnosis of cancer without any side effects, polymeric colloidal materials should have the following qualities:

- Smaller in size
- Eco-friendly
- Low toxicity to ordinary cells
- High stability in biological conditions
- Capable of conveying the imaging agents
- Releasable for therapeutic agents easily

Gadolinium-based nanoparticles have been used for the detection of cancer in magnetic resonance imaging (MRI) due to better contrast. Boyes et al. [115] reviewed molecular probes’ development of gadolinium-based nanoparticles and their importance in cancer therapy. Likewise, Cao et al. [116] discussed the importance of gadolinium-based nanoparticles in the detection of cancer. It was described that the nanoparticles may be hopeful for better longitudinal reflexivity of ions of gadolinium. Moreover, the progress and uses with imminent outlooks were highlighted for their development in the future. Mi et al. [117] synthesized chelates of gadolinium to be loaded as nanocarriers such as MRI-guided gadolinium neutron capture therapy (GdNCT) of lumps. The research group established Ca**_3_**(PO**_4_**)**_2_** micelles attached with anionic copolymers and combined with magnetic resonance imaging contrast agent Gd-DTPA/CaP (gadolinium-diethylenetriaminepentaacetic acid). The contrast agent showed an improved spreading of gadolinium-diethylene triamine penta-acetic acid in cancers. Vuu et al. [118] prepared gadolinium-rhodamine-based nanoparticles for the detection of cancer. For the preparation of such nanoparticles, a lipid was used as a monomer. Moreover, according to the authors, the prepared nanoparticles could be used in the tracking of cells in vivo.

Other works in the track of diagnosis of cancer are defined in the subsequent sub-divisions. Mitra et al. [119] discussed the applications of colloidal particles such as micelles for the diagnosis of cancer. The authors found that positron emission tomography [PET] computed tomography; having nuclear imaging techniques such as dual-modality; could be used in the detection of cancer in various models. Furthermore, it must be remembered that all data regarding the toxicity and other clinical parameters should be studied before smearing in problems of real life. Park et al. [120] and Janib et al. [121] found that polymeric colloidal particles including micelles can also be used for cancer diagnosis. Many inventions were useful for drug carriers. Poly (ethylene glycol) and ATP aptamer were used by Dong et al. [122] not only for the detection of breast cancer but also for the ATP level estimation in the biotic trials. The reported limit of detection was 0.1 pM having a 0.1–1000 pM linear range. Gold-based nanoparticles and micelles attached to SPION were used by Sun et al. [123] for the detection of brain cancers. These nanoparticles played an important role in computed tomography and MRI as contrast agents. The data were used in the determination of tumor size. The authors also reported these as excellent nanoparticles in MRI even after a few days. In addition, the other importance of the synthesized nanoparticles was also confirmed in terms of the detection of early brain cancer. Guo et al. [124] used polymeric nanostructure (PHEMA-star-PLLA-PEG) for the diagnosis of cancer. Substances such as 1,4,7-triazacyclononane, 1,4,7-triacetic acid, and TRC105 were grafted on the end groups for CD105 and ^64^Cu tagging followed by PET imaging.

In the MRI, the nanoparticles with magnetic properties have also played an important role because they offer better contrast. In this series, Li et al. [125] prepared Fe_3_O_4_ nanoparticles coated with aqueous dispersive polyethyleneimine (PEI). After that, nanoparticles were modified with PEGylated folic acid (FA) and fluorescein isothiocyanate (FI) with the help of conjugation of PEI facilitation. For the formation of colloidally stable nanoparticles, the acylation of the residual PEI surface amines was done. The obtained nanoparticles were functionalized with folic acid so that Fe_3_O_4_ nanoparticles could be used for MRI. It was also claimed that the developed nanoparticles may be very helpful in the diagnosis of various cancers. Wang et al. [126] synthesized the optically triggered nanoparticles for cancer diagnosis. The nanoparticles were synthesized using perfluorohexane liquid and gold nanoparticles. After that, a poly (lactide-co-glycolic acid) (PLGA) polymer was used to stabilize them. The potential of photoacoustic imaging of the synthesized nanoparticles was also confirmed so that they can be used for cancer therapy in the future. Huang et al. [127] discussed nanoparticles based on the polymeric substrate to detect cancer through a simulation study. The most important thing to be noted was the diverse acceptance of nanoparticles by diverse cells (cancer cells as well as normal ones). The reason behind it was the interactions between nanoparticles cell-specific and nanoparticles polymer non-specific. Cancer detection was improved by studying the consequence of the ligand, polymer, and density. In biomedicine, the study could convey appreciated ideas and the suggestion of nanomaterials based on functionalized substrates.

**Figure 6:**
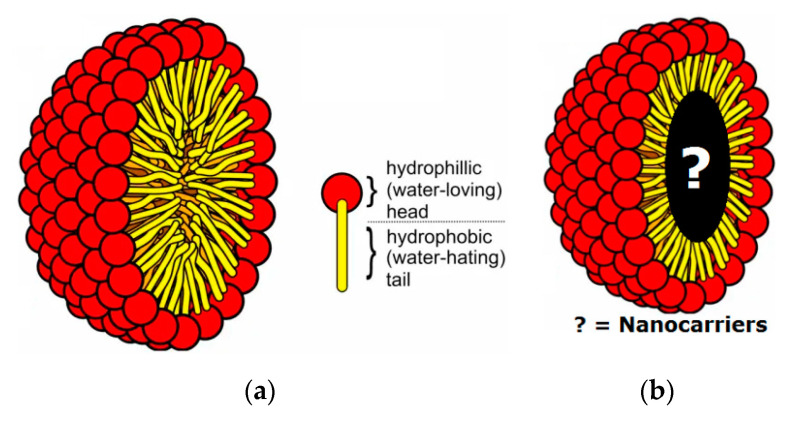
Structure of (**a**) micelle and (**b**) encapsulated nano-carriers.

Sun et al. [123] prepared gold and micelles-loaded polymeric iron oxide nanoparticles for the detection of brain tumors. In addition, these particles were also used as a contrast agent in the study of stereotactically implanted GBM tumors in a mouse model. Based on the results, it could be concluded that the synthesized polymeric nanoparticles might be used in brain tumor treatment, as no side effect was observed during diagnosis. In addition, the micelles with polymeric nature have been used in many cancer diagnostic tools such as X-ray, nuclear imaging, and CT scans MRI [128]. In addition, the micelles in conjugation with ^99m^Tc and ^111^In (emitters of gamma rays) have been used for non-invasive bio-distribution [129,130]. The addition of particles of iron oxide in micelles and the addition of chelators for the metallic blending to the micelle with a hydrophilic wedge are the two approaches for the preparation of MRI based on micelles [131,132]. The magnetic polymeric micelles with multi-functions and imaging differences have been described the effective MRI scanning [133]. Nasongkla et al. [133] described the targeting ligands in chemotherapy as useful agents. Qiao and Shi [134] and Yang et al. [135] discussed the synthesis of nanoparticles based on iron oxide. All those nanoparticles were not only conjugated with Arg-Gly-Asp but also modified with dendrimers for the targeted MRI scanning for C6 glioma cells as shown below in Figure 6.

## 5. Benefits of Polymeric Colloidal Material in Cancer Treatment

In the present time, nanotechnology is gaining importance in many areas of research including cancer diagnoses and treatments. Although different types of nanoparticles have been used to control cancer yet the nanoparticle-based polymeric colloidal materials are taking place deep in the treatment of cancer. The reason behind it is biocompatibility, non-toxicity, non-immunogenicity, and biodegradability. The nanoparticle-based polymeric colloidal materials are being used in the treatment of cancer continuously. The important features of these in the treatment of cancer are described below. The nanoparticle-based polymeric colloidal materials should exhibit the following:

- biocompatibility, non-toxicity, non-immunogenicity, and biodegradability.
- transport essential drugs.
- release the medicines at the tumor location.
- be stable in physiological conditions.
- control the effect of EPR or receptor-facilitated interfaces.

The nanoparticles based on the polymer are outstanding transporters of essential drugs in the treatment of cancer. Many laboratories synthesized precursors, as well as nature-based precursors, which have been used to prepare nanoparticle-based polymeric colloidal materials. The laboratories synthesized precursors contain polylactic-co-glycolic acid, polylactic acid, and the polymer of ethyleneimine while chitosan, collagen, albumin, and gelatin are the natural ones. To the best of our knowledge, the first nanoparticle-based polymer was used by Couvreur et al. [136] for the treatment of cancer. The polymer of alkyl cyanoacrylate was used as raw material for the preparation of the nanoparticle-based polymeric colloidal material. After this innovation, many nanoparticle-based polymeric colloidal materials were synthesized to cure cancer; particularly anti-cancer drug delivery. The advantages of the nanoparticle-based polymeric colloidal materials in the treatment of cancer are deliberated as follows.

### 5.1. Ecological

The recyclable nanoparticle-based polymeric colloidal materials are submicron in size. The nanoparticles preparation is performed in two ways: (i) by allocating the achieved polymers and (ii) by monomeric polymerization [137]. These nanoparticles are informal to show biocompatibility, non-immunogenicity, non-toxicity, and water-solubility at low cost. These have played an important role as an operative process for supplying drugs to a definite tissue of organs, as a means of not only gene therapy but also DNA therapy, and in their fitness to allot proteins and genetic factors by verbal administration [138]. Many anti-cancer drugs such as doxorubicin, paclitaxel, 5-fluorouracil, cisplatin, 9-nitrocamptothecin, triptorelin, dexamethasone, and xanthone, etc. are being found efficiently associated with the polymer of glycolic acid [139]. The spheres with nanoscale have a decomposable polymer of caprolactone showing hydrophobicity [140]. In addition, they also have a decomposable methoxy polymer of ethylene glycol showing hydrophilicity which is used for the delivery of taxol.

### 5.2. Polymeric Micelles

An associated colloid is termed a micelle that has both hydrophilic and hydrophobic moieties. Because of the presence of both moieties, self-assembling takes place and a hydrophobic center with a constant hydrophilic encapsulation is molded. When these polymeric micelles are used as drug carriers, they are not only more soluble but also able to easily approach tumors. The self-assembling property is the main cause of the encapsulation of the drugs which helps too much in carrying the drugs to their target. The collection of them is conducted by diverse methods including the preparation of monomers in the main core [141,142]. The micelles are identified as the transporters of colloid for hydrophobic drugs [143,144]. Hence, they have decent chemotherapeutic characters [126,144]. The smaller size of the micelles makes them penetrable in a tumor. Due to this, the release of the drugs takes place easily. To the best of our knowledge, the use of polymeric micelles as the drug carrier was clinically approved in South Korea [145]. Paclitaxel preparation has received FDA approval to be used in the treatment of breast cancer [126]. The literature survey shows some reviews written regarding the applications of micelles in the delivery of drugs [47,146]. Valenzuela-Oses et al. [147] reported an invention related to polymeric micelles loaded with miltefosine. The anti-cancer activity of the formulation was checked against HeLa cell lines, which displayed hopeful outcomes. Thermal analyses indicated the spreading of miltefosine. Miltefosine (80 μM) in combination with micelles of pluronic-F127 polymer presented a notable reduction in hemolytic outcome in divergence to free drugs. The micelles based on pluronic-F127 polymer loaded with miltefosine could be an advantage in nanocarriers-based tumor therapy. Yang et al. [148] synthesized a polymer of ethylene glycol derivatized with GA for the delivery of DOX. The formulation showed a real synergistic effect on apoptosis and inhibition of cell proliferation. In addition, a long time of blood circulation, low supply, and DOX release were also observed in the case of the proposed formulation. The bio-distribution studies showed the accumulation of the drug at the tumor site. The so-formed micelles-based prodrugs could be co-delivered to the target to achieve the action of gambogic acid and DOX. Wang et al. [149] used ethylene glycol and ε-caprolactone and trimethylene carbonate to improve the blood circulation of gambogic acid followed by its accumulation at the tumor site. Volsi et al. [150] synthesized polymer-based micelles and gold core-shell quantum dots. The authors used these for the encapsulation of doxorubicin. As per the authors, the reported formulation had good anti-cancer activities for breast cancer.

### 5.3. Miscellaneous Functions

Nanoparticle-based polymeric colloidal materials have also been mixed with other materials for the stuffing and transportation of drugs. These include polystyrene, chitosan, etc. Kim et al. [151] described chitosan nanoparticles being used for the controlled release of the drugs. Deformability, constancy, and fast interest by cancerous cells were also described. Paclitaxel was the loaded drug, which showed good activity with low uptake by the normal cells. Park et al. [152] used cholanic acid for the derivatization of glycol chitosan to increase the anti-cancer activity of the synthesized polymeric nanoparticles. This derivatized material showed better tumor accumulation, satisfactory dispersal, and time-dependent elimination in SCC7 tumors. Near-infrared fluorescence was used to shoot images. In addition, other characteristics regarding the derivatized materials were long-reduced time-dependent elimination, time of blood circulation, and higher tumor accumulation with the increase in molecular weight of the polymer. The noticeable point was a long time resting in the blood with an increase in molecular weight of the synthesized polymeric nanoparticles based on glycol chitosan. Due to the increased concentration of blood, an increased accumulation at the site of cancer was observed. In this way, the authors improved not only the blood circulation period but also the cancer-targeting ability of the synthesized polymeric nanoparticles. Polymeric micelles were used by Gao et al. [153] for the encapsulation of doxorubicin. Pluronic P-105 and PEG2000 diacyl phospholipids were used as raw materials for the preparation of nanoparticle-based polymeric colloidal materials, while the polymer of ethylene glycol and beta-benzyl-L-aspartate were used in not only micelles formation but also as carriers of the drugs. A comparative study between the bio-distribution of the synthesized drug and the molecularly softened one in the tumor of the ovary was done. Better results were observed in the case of bio-distribution in the formulation based on encapsulation. Lammers et al. [154] used *N*-(2-hydroxypropyl)-methacrylamide polymer and radionuclide (^131^I) for cancer treatment. In addition, gemcitabine and doxorubicin were also loaded to improve the efficiency of therapy. In the experiment, a selective assembly and lengthy period of the movement were indicated by the radioactive transferor of the polymer. The satisfying effects on tumors were increased by the synergistic effect.

The most important advantages of nanoparticle-based polymeric colloidal materials are their smaller size, biocompatible nature, biodegradable nature, low poisonousness to normal cells, extraordinary constancy in biological circumstances, capability to convey the imaging mediators, and precise discharge of medicines. In addition, the ease of their synthesis with decent control over the size and spreading may be considered as another advantage. Additionally, the nanoparticle-based polymeric colloidal materials with encapsulated drugs are safe from an environmental point of view. During the write-up of this article, it was realized that there is no side effect associated with nanoparticles of the polymeric colloids. However, the imperfect pointing capabilities and the problem of therapeutic termination may be measured as the chief disadvantages. An imbalance in disruption may happen automatically via nanoparticle-based polymeric colloidal materials. Rarely, nanoparticle-based polymeric colloidal materials also affect the function of the heart [155].

## 6. Future Perspectives

During the write-up of this article, it was realized that polymeric colloidal materials are important in cancer diagnosis and treatment due to the controlled release of the drugs, stability to labile molecules, and ease of surface modification for targeted drug delivery. The biological applications increased many time over when these materials are in nano size [156]; however, it is important to mention here that the research on polymer colloids have been going on for about three decades but still, these materials are not explored fully due to certain limitations such as the desired tenability in size (especially in nano size), and the stability in the blood. Sometimes, the drug diffusion or early release of the loading drugs may be a problem with these materials. Sometimes, the polymer colloids, especially the liposomes, are destroyed through the phagocytosis phenomenon. This can be delayed or avoided by using Steaith^TM^ liposomes. The lack of specific targeting technology is one of the most serious hurdles to the success of the polymer colloids’ role in cancer treatment. Therefore, it is urgently required to prepare magnetic polymer colloids to control the drug release via magnetic effect. Such polymer colloids may be prepared by anchoring some magnetic nanoparticles of the various metal ions. A better quality understanding of the physiological restrictions; controlling the fate and distribution of these carriers; has permitted for more rational design and the expansion of advanced generation systems. We found only a small number of papers on this topic as the research is in progress. In this respect, there is a big demand to work for cancer diagnosis and treatment using colloidal polymeric materials.

## 7. Conclusions

Despite the great efforts of the researchers, the chemotherapy strategy of cancer treatment is not completely safe and human-friendly. Many approaches, such as small molecule inhibitors, nanomedicines, etc., have been developed but still chemotherapy needs more research and attention. Polymer colloids are a very important class of materials due to their unique features. They have a wide range of applications and may be used to anchor anti-cancer drugs for safe chemotherapy. The polymer colloids are important to drug carriers as they can protect drugs from biodegradation in the blood stream. In addition, the polymer colloids deliver an ultra-dispersed form of the drugs without using annoying solvents and allow fast drug dissolution. Consequently, it is supposed that polymer colloidal can increase the efficiency of the actions of anti-cancer drugs. In this article, the utility of these materials (micelles, liposomes, emulsions, cationic carriers, and hydrogels) is discussed in cancer diagnosis and treatment. Some polymer colloids-based anti-cancer drugs are under clinical trial. Hopefully, improved drugs that comply with the requirements will soon receive approval in clinical practice. Most of these materials have been used to anchor the drugs for release at the targeted points. Still, there is a need to make magnetic polymer colloids more useful; especially by derivatization to anchor anti-cancer drugs. This may lead to a good working of polymer colloids in a varied range of pHs and temperatures. Briefly, it is concluded that polymer colloids have a wide range of scope in cancer diagnosis and treatment. We hope that in the future these materials will provide a wider range of choice in cancer diagnoses and treatments.

## Figures and Tables

**Table 1:** The micelle-based materials act as nanocarriers.

Class	Polymeric Unit	Combining Units	Obtained Skeleton	Refs.
Pluronics	poly(propylene oxide)	propylene oxide and ethylene oxide	(PEO)a-(PPO)b-(PEO)a	[34]
	None	Poloxamer and MTX	p-MTX	[40]
	TPGS (D-α-tocopheryl polyethylene glycol Succinate)	Poloxamer 427 and 27 and vitamin E TPGS	PLGA-TPGS/Poloxamer 235	[41]
PEG	PLA	Dodecanol, folic acid and PEG-PLA	Dol-PLA-PEG-FA	[43]
	PLA	Ala-Pro-Arg-Pro-Gly and	peptide-maleimide-PEG-PLA	[44]
	acid-chloride of PCL	PCL-COCl, PEG and TAPC	mPEG-b-PCL copolymer	[45]
	PCL	PEG-PCL and folic acid	Folate-PEG-PCL	[46]
	poly(ethylene glycol)-distearoyl phosphoethanolamine	Lipofectin and PEG-PE	Lip-PEG-PE	[47]
	poly(ethylene glycol)-distearoyl phosphoethanolamine	PEG-PE, Lipofectin lipids and paclitaxel	PEG-PE/ST/LL	[48]
	PLGA	PLGA, PEG, Dox and nanoparticle	Dox-NP	[49]

**Table 2:** Liposomal-based antiicancer drugs [57].

Name of Drugs	Status	Compony
Doxorubicin	Doxil	Sequus (Alza)
Daunorubicin	DaunoXome^TM^	NeXstar (Gilead Sciences)
Edolfosine	Phase I	The Liposome Company
Tretinoin	Phase I/II	Aronex
Cisplatin	Phase II	Aronex
Annamycine	Phase I/II	Aronex

**Table 3:** The polymer colloids-based anti-cancer drugs in clinical trials.

Drugs	Grade	Tumor types	Refs.
Paclitaxel	Accepted	Lung, breast, and pancreatic	[74]
Rapamycin	Under clinical trial	Solid	[75]
Docetaxel	Under clinical trial	Prostrate and breast	[76]
Doxorubicin	Under clinical trial	Hepatocellular	[77]
Mitoxantrone	Under clinical trial	Hepatocellular	[78]
Docetaxel	Under clinical trial	Solid	[79]
Paclitaxel	Under clinical trial	Neoplasms	[80]
DACHPt	Under clinical trial	Ovarian	[81]

DACHPt: Dichloro(1,2-diaminocyclohexane)platinum(II).

**Table 4:** FDA-approved nanomedicines for the treatment of cancer [111].

Materials	Names	Indication	Year(s) Approved
Liposome-PEG	Doxorubicin	Metastatic breast cancer, metastatic ovarian cancer	1995
PLGA	Leuprolide acetate	Prostate Cancer	2002
Albumin	Nab-paclitaxel	Metastatic breast cancer	2005
		Pancreatic cancer	2013
mPEG-PLA	Paclitexal	Metastatic breast cancer	2007
Liposome	Lrinotecan	Pancreatic cancer	2015

## Data Availability

Not applicable.
